# Exploring Functionalized Magnetic Hydrogel Polyvinyl Alcohol and Chitosan Electrospun Nanofibers

**DOI:** 10.3390/gels9120968

**Published:** 2023-12-11

**Authors:** Mónica Guerra, Fábio F. F. Garrudo, Célia Faustino, Maria Emilia Rosa, Maria H. L. Ribeiro

**Affiliations:** 1 Faculty of Pharmacy, Universidade de Lisboa, Av. Prof. Gama Pinto, 1649-003 Lisboa, Portugal; guerramsc@gmail.com (M.G.); cfaustino@ff.ulisboa.pt (C.F.); 2 Department of Bioengineering, Institute of Telecomunications, Instituto Superior Técnico, Universidade de Lisboa, Av. Rovisco Pais, 1049-001 Lisboa, Portugal; fabio.garrudo@tecnico.ulisboa.pt; 3 Research Institute for Medicines (iMed.ULisboa), Faculty of Pharmacy, Universidade de Lisboa, Av. Prof. Gama Pinto, 1649-003 Lisboa, Portugal; 4 Department of Pharmaceutical Sciences and Medicines, Faculty of Pharmacy, Universidade de Lisboa, Av. Prof. Gama Pinto, 1649-003 Lisbon, Portugal; 5 Instituto de Engenharia Mecânica (IDMEC), Instituto Superior Técnico, Universidade de Lisboa, 1049-001 Lisboa, Portugal; emilia.rosa@ist.utl.pt

**Keywords:** lysozyme, 3D-electrospun, hydrogels, chitosan, PVA, magnetic nanoparticles, tunable properties

## Abstract

Nanofibrous materials present interesting characteristics, such as higher area/mass ratio and reactivity. These properties have been exploited in different applications, such as drug-controlled release and site-specific targeting of biomolecules for several disease treatments, including cancer. The main goal of this study was to develop magnetized nanofiber systems of lysozyme (Lys) for biological applications. The system envisaged electrospun polyvinyl alcohol (PVA) and PVA/chitosan (CS) nanofibers, loaded with Lys, crosslinked with boronic acids [phenylboronic acid (PBA), including 2-acetylphenylboronic acid (aPBA), 2-formylphenylboronic (fPBA), or bortezomib (BTZ)] and functionalized with magnetic nanobeads (IONPs), which was successfully built and tested using a microscale approach. Evaluation of the morphology of nanofibers, obtained by electrospinning, was carried out using SEM. The biological activities of the Lys-loaded PVA/CS (90:10 and 70:30) nanofibers were evaluated using the *Micrococcus lysodeikticus* method. To evaluate the success of the encapsulation process, the ratio of adsorbed Lys on the nanofibers, Lys activity, and in vitro Lys release were determined in buffer solution at pH values mimicking the environment of cancer cells. The viability of Caco-2 cancer cells was evaluated after being in contact with electrospun PVA + Lys and PVA/CS + Lys nanofibers, with or without boronic acid functionalation, and all were magnetized with IONPs.

## 1. Introduction

Nanofibers can be produced from a wide range of natural and synthetic polymers. Some of the natural polymers include hydrogels such as alginate (AL), chitosan (CS), collagen (CO), gelatin (GE), fibrin (FI), and hyaluronic acid (HA) [1,2]. CS, a natural polysaccharide obtained from the deacetylation of chitin, has found widespread use in the pharmaceutical, food, and biotechnology industries due to its biodegradability, biocompatibility, and biological properties, such as antioxidant, and antibacterial properties [3]. In the field of biomedicine, chitosan gel has been employed as a polymeric component in drug delivery, bone tissue regeneration, and the healing of skin lesions [4]. Additionally, it is also appropriate to remark on the possibility of combining chitosan with other types of hydrogels to create suitable materials for improved tissue engineering applications [5].

Synthetic polymers such as poly(vinyl alcohol) (PVA), poly(hydroxyethyl methacrylate), poly(ethylene glycol dimethacrylate), poly(ethylene oxide), poly(propylene-co-ethylene glycol fumarate), polypeptides, and poly(acrylic acid) and its derivatives have also been used to produce nanofibers. PVA is an electrospinnable hydrophilic gel with excellent mechanical properties, biodegradability, non-toxicity, and biocompatibility. It has an acceptable toxicological profile, with LD50 above 15–20 g/Kg, NOAEL of 5 g/Kg, low gastrointestinal absorption, lack of accumulation in the body, no sub-chronic or chronic toxicity events reported, and no mutagenic or carcinogenic effects observed on in vitro assays [6]. PVA is considered a safe compound by the European Food Safety Agency (EFSA) and the Food and Drug Administration (FDA). Owing to its properties, PVA is one of the most extensively studied and widely used polymers in biomedicine, especially in contact lenses, implants, drug delivery systems, tissue engineering, artificial organ development, and immobilization [2].

Polymer blending has emerged as a method of enhancing the chemical and mechanical properties of polymeric materials for practical applications [7,8,9]. The existence of electrospinning emulsions of poly (L-lactic acid)/poly (vinyl alcohol) with chitosan were used for wound dressing with antibacterial properties [10]. Electrospinning is one of the most popular processes for producing nanofibers, and it has been of interest since the 1980s, inspired by the development of nanotechnology. Electrospinning allows for the conversion of a polymeric solution into solid nanofibers via the application of electrical force [11,12]. The nanofibers obtained using electrospinning are collected in the form of a porous matrix with a high surface area, which is structurally similar to the extracellular matrix [11,12]. These fibers are associated with a low production cost and simplicity of manufacturing, making them a promising substrate with vast applicability in numerous areas of chemistry, biology, medicine, and engineering, such as wound healing, wound care, biosensors, drug delivery systems, medical implants, tissue engineering, dental materials, filtration membranes, protective clothing, and other industrial applications [11,12].

Lysozyme is a widely distributed enzyme found in several organisms, such as bacteria, bacteriophages, fungi, plants, and mammals [13,14]. Chicken egg white lysozyme, which has 129 amino acids and reactivity 3–4 times lower than human lysozyme, has been widely used as an experimental model due to its structural similarity, availability, and low cost, making it one of the most studied enzymes [15].

The antimicrobial activity of lysozyme relies on its ability to catalyze the hydrolysis of the β1-4 glycosidic bond between *N*-acetylmuramic acid and *N*-acetylglucosamine, which are components of the peptidoglycan in bacterial cell walls [16].

Lysozyme’s bactericidal properties have been applied in the food and pharmaceutical industries [17]. In addition to its bactericidal properties, antifungal, antiviral, antitumor, and immunomodulatory properties have also been described [13]. Lysozyme’s association with cancer began in the 1960s due to its role as a tumor biomarker in hematological cancers [6,18].

In recent years, the immobilization of enzymes and magnetic nanoparticles on polymeric nanofibers has allowed their use in sensors, tissue regeneration structures, drug delivery systems, and other applications [19]. Iron oxide nanoparticles (IONPs) have paramagnetic properties, are biocompatible and non-toxic, and are suitable for biomedical applications.

A different approach was the development of co-immobilized cellulase and lysozyme on the surface of amino-functionalized magnetic nanoparticles using glutaraldehyde [20]. Another approach [21] was the fabrication of magnetic lysozyme@Fe_3_O_4_ composites via amyloid-like assembly for uranium extraction with magnetism for easy recovery and good binding affinity towards uranium, respectively. These composite adsorbents also showed excellent photothermal properties derived from the Fe_3_O_4_ nanoparticles.

A 2D protein self-assembly film was reported to capture functional enzymes without any further chemical modification, with enzymes immobilized between Fe_3_O_4_ nanoparticles and a lysozyme film, preventing enzyme leaching and ensuring contact with substrates [22,23].

To extend the lifespan and bioactivity of lysozyme for use in food packaging, medicine, medical devices, and cosmetics, immobilization of lysozyme on solid supports showed positive results, as demonstrated by the increased stability and extended half-life of the enzyme [24].

Magnetic nanoparticles have gained widespread research interest due to their additional use in hyperthermia-based cancer therapy. The process is based on the increased sensitivity of various types of cancer cells to temperatures above 41 °C. Iron oxide nanoparticles (IONPs), such as magnetite (Fe_3_O_4_), can heat the surrounding environment to 45 °C when an alternating magnetic field is applied to them (hyperthermia). The damage caused by the application of temperatures in the range of 41–45 °C in normal tissue is reversible, while tumor cells are irreversibly damaged, and cell death occurs [19,25]. Therefore hydrogel nanofibers magnetized with IONPs can be one strategy for tumor treatment based on localized hyperthermia [19]. The use of magnetized scaffolds as a therapeutic system and as a drug delivery system is an interesting approach to colon cancer therapy. The magnetic nanoparticle properties directly rely on their morphology and size. Thus, as the nanoparticle size decreases, the magnetic behavior of the particle enormously decreases [26], which directly impacts their applications as drug carriers or in hyperthermia treatments. Colon cancer is the third most common cancer worldwide and the fourth most common cause of death [27]. Due to its invasive nature, there is a need for alternative colon cancer therapies, especially ones that allow growth control, enclosure of metastatic cells, and recurrence. The human epithelial cell line Caco-2, derived from colon carcinoma, has been widely used as a model of the intestinal epithelial barrier [28,29,30] and was used in this study to test the cytotoxicity of the developed lysozyme magnetized nanofibers. Therefore, the main goal of this study was to develop a hydrogel nanofiber system using the electrospinning technique, with the encapsulation of lysozyme crosslinked with boronic acids and magnetized with IONPs.

## 2. Results and Discussion

The morphology of nanofibers depends on several factors, namely, properties of the polymeric solution (concentration, molecular weight, viscosity, conductivity, surface tension), process parameters (voltage, flow rate, collectors, distance between collector and syringe needle), and environmental conditions (humidity and temperature) [31].

### 2.1. Characterization of Electrospinning Solutions

**Figure 1:**
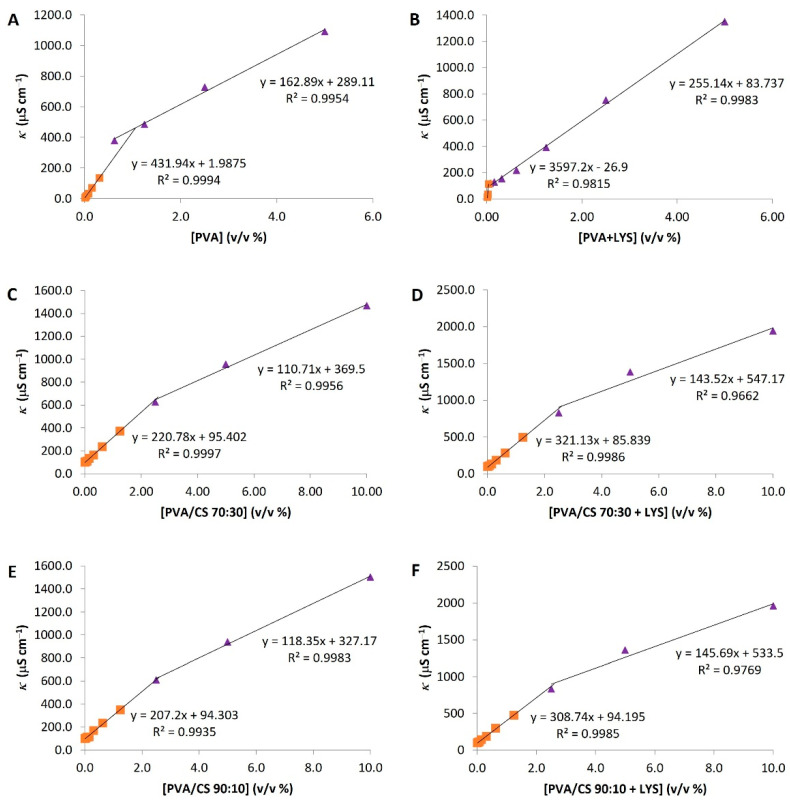
Specific conductivity (average temperature of 18.5 °C). (**A**) PVA; (**B**) PVA + Lys; (**C**) PVA/CS 70:30; (**D**) PVA/CS 70:30 + Lys; (**E**) PVA/CS 90:10; (**F**) PVA/CS 90:10 + Lys. SD (Standard Deviation) was ±0.01, and each point of the graphic was carried out in triplicates.

The specific conductivity (κ) was evaluated for the hydrogel solutions of PVA, PVA + Lys, PVA/CS 90:10, PVA/CS 90:10 + Lys, PVA/CS 70:30, PVA/CS 70:30 + Lys, and CS. Figure 1 presents the specific conductivity profile as a function of polymer concentration. For CS, no conductivity profile was observed.

The specific conductivity increased linearly with hydrogel polymer concentration up to the point of discontinuity (Figure 1). This profile was divided into two linear series that were adjusted for each one and allowed the critical aggregation concentration (c.a.c.) calculation for each solution based on the intersection of the two lines (Table 1). These results suggest that both Lys and CS positively influence the conductivity values of the tested solutions. Similarly, the contribution of the CS to the c.a.c. represents an increase of about 1.5% in the concentration required for the 10% PVA solution to reach the c.a.c. (Table 1). Moreover, Lys in PVA/CS solutions increased the c.a.c. values. On the contrary, for the PVA + Lys solution, the conductivity values increased proportionally with the concentration, with a cutoff point at 0.03% PVA concentration (Figure 1).

**Figure 2:**
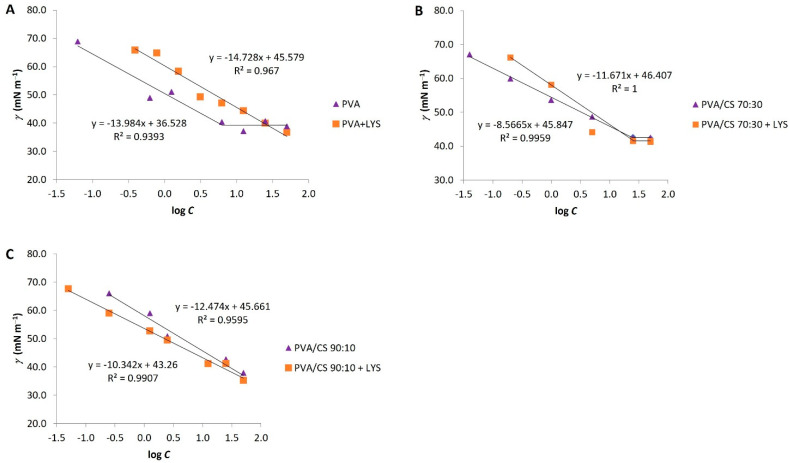
Surface tension profiles as a function of the logarithm of polymer concentration for PVA, PVA + Lys solutions (**A**); PVA/CS 70:30, PVA/CS 70:30 + Lys (**B**); and PVA/CS 90:10, PVA/CS 90:10 + Lys (**C**). SD (Standard Deviation) was ±0.01, and each point of the graphic was carried out in triplicates.

The surface tension (γ) was another parameter used in the characterization of the electrospinning solutions. Figure 2 represents the surface tension profiles as a function of the logarithm of polymer concentration. The surface tension of the solutions decreased with increasing concentration of the polymers, attaining an equilibrium at the point where the c.a.c. was achieved for PVA solutions: PVA/CS 70:30 and PVA/CS 70:30 + Lys. The presence of CS did not significantly alter the surface tension values; however, the PVA/CS 70:30 solutions originated higher values. Moreover, the presence of Lys decreased the surface tension values at c.a.c. of all analyzed solutions (Figure 2).

The c.a.c. was calculated based on surface tension data and fitted for conductivity data (Table 1). In this case, the values of c.a.c. are similar for all solutions except for the 10% PVA + Lys solution, which showed some variability between the results calculated from the conductivity and surface tension.

An increase in the conductivity of the solution promotes the formation of thinner fibers, contrary to the surface tension, which should not be too high, as it directly influences the shape of the formed structures, fibers, or drops.

It was not possible to produce nanofibers from the 2% CS gel solution despite having a higher conductivity (2324 S cm^−1^) than the PVA/CS gel solutions. The results suggest an increase in this parameter by mixing the PVA/CS gel polymers. In the various attempts to produce fibers with this solution, the phenomenon of electrospray can be attributed to the low concentration of the gel polymer and a probable increase in surface tension. This gel solution was less viscous than the other solutions tested, which may hinder the production of nanofibers.

### 2.2. Morphology of the Nanofibers

The morphology of nanofibers is influenced by several parameters during the production process, namely temperature and humidity conditions, voltage, solution flow rate, the distance between the capillary end and the collector, and, in particular, the properties of the polymer(s) solution, including the concentration, viscosity, conductivity, surface tension, and nature of the solvent.

Not all attempts to produce PVA or PVA/CS nanofibers were effective, with the temperature of the electrospinning solution being the main parameter, with a consequent influence on viscosity. Thus, the optimal temperature range for electrospinning was 18–20 °C.

In the electrospinning, the gel solutions of 2% CS in 2% acetic acid, 2.5% CS in 50% acetic acid, and 4% and 3% CS in 90% acetic acid were tested, but in none of the cases, nanofibers were obtained. The production of pure CS nanofibers was very difficult due to the sensitivity of the process to humidity (<30%). However, the electrospinning process was improved by introducing the other gel polymer, PVA, and tested in different proportions (90:10 and 70:30).

The nanofibers obtained with PVA or PVA/CS, with and without Lys, presented, for the most part, a uniform appearance, white color, relatively thin and fragile, with a circular shape with a diameter of about 3.3 cm and a mass mean of 4.12 mg (Supplementary Material, Figure S1). Some reported studies where homogeneous fibers were obtained when chitosan was mixed with synthetic resins and nanoparticles to strengthen the gathered results [32].

**Figure 3:**
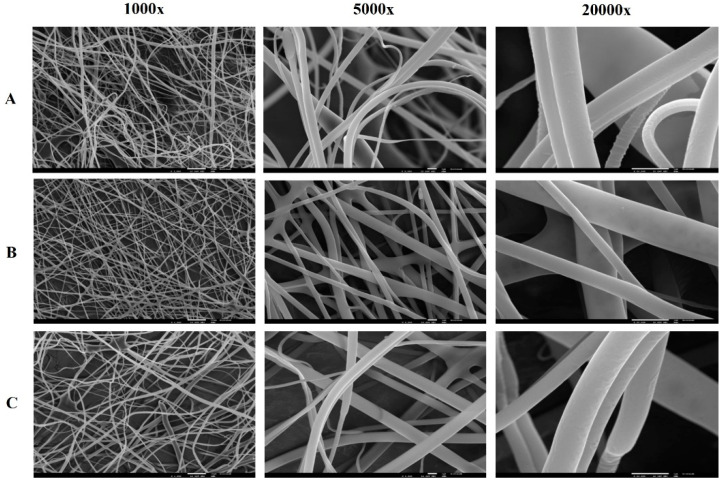
SEM images of the nanofibers of PVA/CS 70:30 (**A**); PVA/CS 90:10 (**B**), and PVA/CS 90:10 + Lys (**C**), magnification of 1000×, 5000× and 20,000×.

PVA and PVA/CS nanofibers with different treatments were observed by optic microscopy and SEM with different magnifications (Supplementary Material, Figure S2). Figure 3 shows the images of PVA and PVA/CS nanofibers using SEM, respectively.

**Figure 4:**
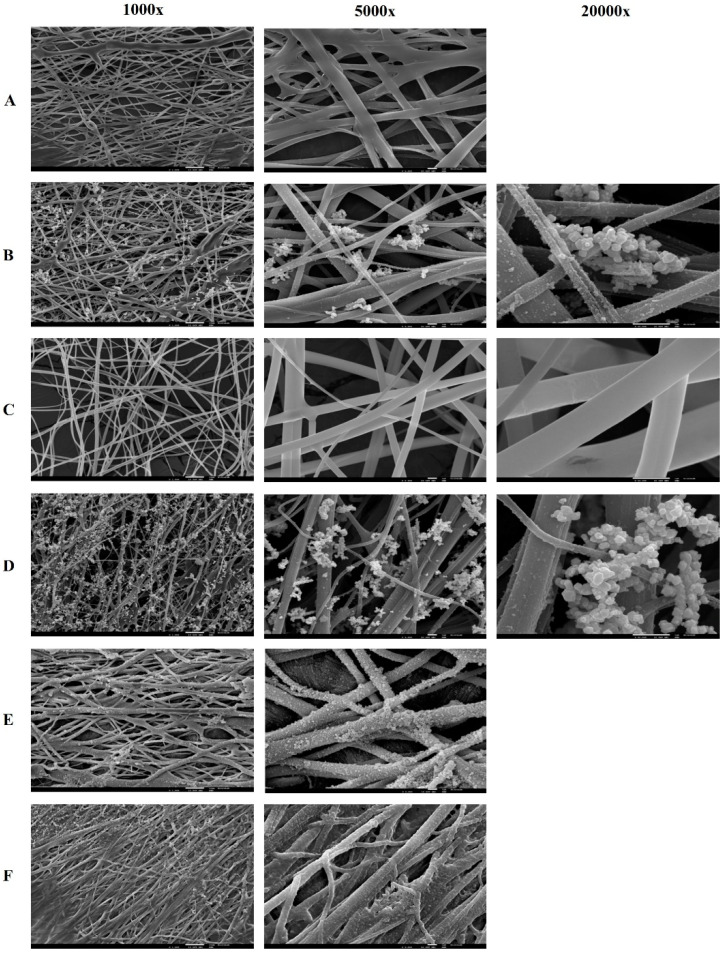
Electron microscopy images of nanofibers of PVA + PBA (**A**); PVA + PBA + IONPs (**B**); PVA + Lys (**C**); PVA + Lys + PBA + IONP (**D**); PVA + Lys + fPBA (**E**); PVA + Lys + aPBA (**F**) at 1000×, 5000× and 20,000×.

The mixture of PVA/CS polymers did not show significant differences in terms of morphology. SEM images show, for both PVA/CS 90:10 and 70:30 ratios, nanofibers with fibers of variable diameters, and some fibers appear folded on the surface, namely the fibers with Lys and in the PVA/CS 70:30 ratio (Figure 4). In terms of fiber diameter, compared with PVA fibers, they show similar diameters; however, there is greater porosity between fibers, especially the PVA/CS 70:30 nanofibers. PVA/CS 70:30 nanofibers also appear to be more flexible than PVA or PVA/CS 90:10 nanofibers.

The conjugation of PVA with CS in the formation of nanofibers seems to improve their chemical and resistance properties [33]. The interactions between these molecules are essentially based on the hydrophobic aggregation of the side chain and intermolecular and intramolecular hydrogen bonds [34].

After crosslinking with boronic acids, the nanofibers become less fragile. The immersion of these nanofibers in suspensions with iron oxide nanoparticles resulted in the sedimentation of black nanoparticles on the fiber, presenting a rough appearance with different colors (Supplementary Material, Figure S1).

All nanofibers produced presented randomly oriented fibers with variable diameters. Through the optical microscopy evaluation, it was observed that the nanofibers with Lys immobilized seem to have some drops on the fibers (Supplementary Material, Figure S2), which translates into roughness in the SEM images (Figure 3). Using optical microscopy, a deposition of brown drops on the fibers that have been crosslinked with boronic acids, namely the acids PBA, aPBA, and fPBA, was observed. SEM images confirm the deposition of inhomogeneous structures on the fibers, making them straighter and thicker, which may be due to the presence of boronic acids (Figure 3). Thus, the influence of these acids on the fibers can be qualitatively inferred based on the fiber diameter, which decreased as follows: fPBA > aPBA > PBA, while pore size decreased with aPBA > fPBA > PBA. The BTZ acid, through observation by optical microscopy, did not affect the nanofibers in a manner similar to the acids described, as it seems to affect the uniformity of the polymeric fibers, destroying their cohesion.

### 2.3. Release and Enzyme Activity Assays

**Figure 5:**
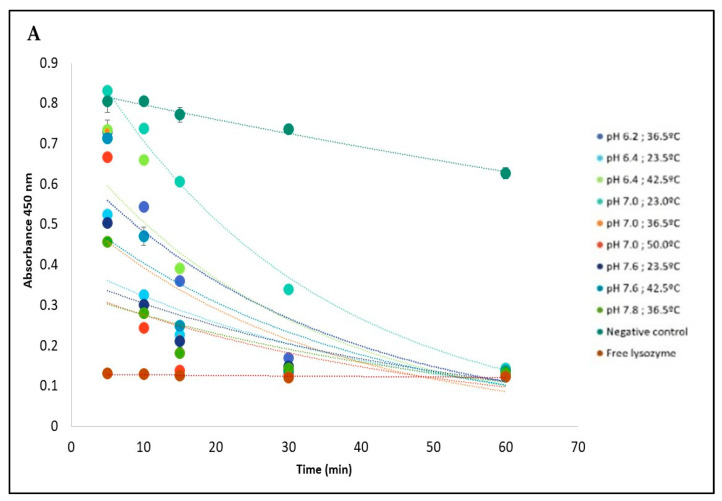

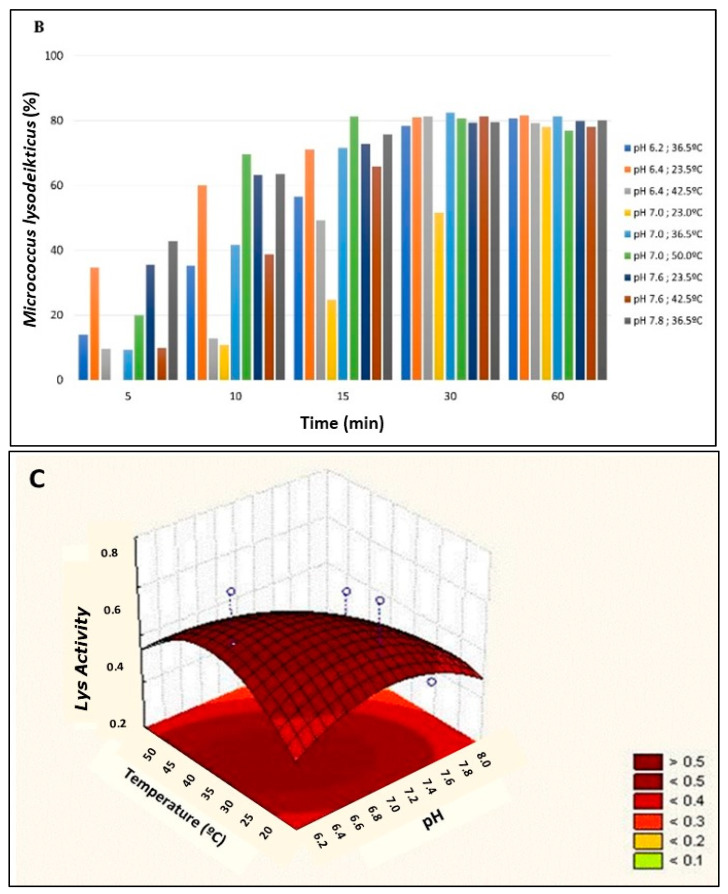
Lys release profile from PVA + Lys + PBA + IONPs fibers based on *Micrococcus lysodeikticus* (3 mg/mL) activity (absorbance at 450 nm) under different pH and temperature conditions (**A**); *Micrococcus lysodeikticus* lysis expressed as a percentage by the difference in absorbances at 450 nm after 60 min (**B**); Results of the CCD matrix to assess the pH and temperature at which lysozyme presented a higher release, based on the microbial reduction of *Micrococcus lysodeikticus* (**C**). SD (Standard Deviation) was ±0.005, and each point and column of the graphics (**A**,**B**) was carried out in triplicates.

The influence of pH and temperature on the release of Lys immobilized on PVA + Lys + PBA + IONPs nanofiber is shown in Figure 5.

Figure 5 shows the absorbance decay at 450 nm corresponding to the activity of *M. lysodeikticus* in contact with the nanofibers for 60 min for the different conditions tested, based on the RSM model. In the case of temperature and pH conditions, the nanofibers showed a controlled release profile compared with the profile corresponding to the free enzyme. After 60 min, a cell lysis rate was equivalent to the positive control with free enzyme (about 80%) (Figure 5B).

Based on the optimized temperature and pH conditions generated from the Response Surface Methodology (RSM) model, it was found that lysozyme showed a high release rate from the nanofiber at pH 6.74 and temperature 45.5 °C (Figure 5C). The model demonstrated excellent suitability as a tool to implement the lysozyme-tailored nanofibers to deliver the enzyme in a potential anticancer application since the pH in the cancer cells is lower than normal cells, and they are sensitive to temperature.

**Figure 6:**
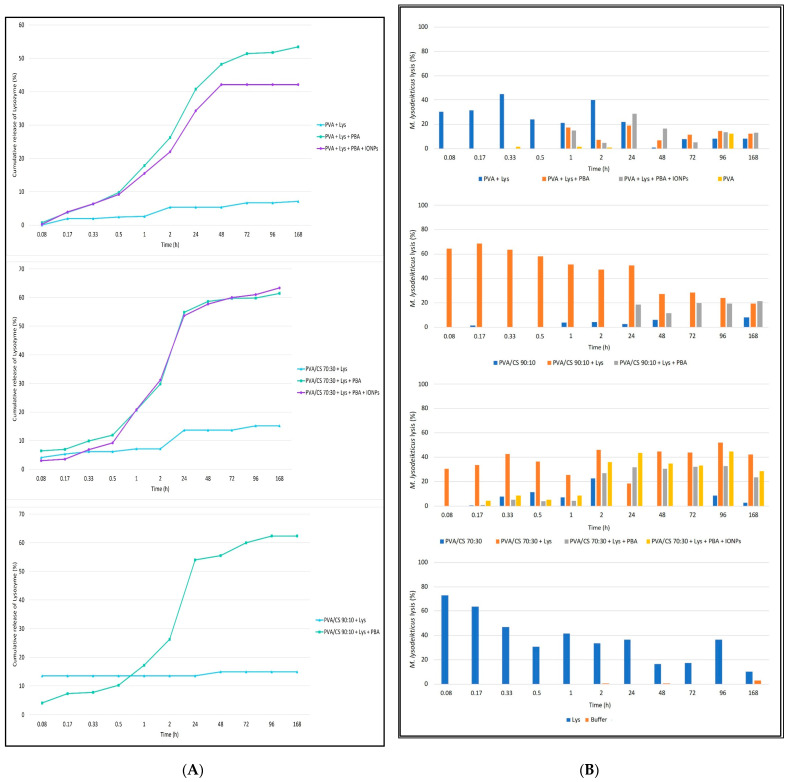
(**A**) Cumulative release profiles of Lys from PVA (1), PVA/CS 70:30 (2), and PVA/CS 90:10 (3) nanofibers. These results are based on the Abs (Absorbance) at 260 nm and the average mass of the nanofibers used (5 mg). All samples were analyzed in triplicate; (**B**) Effect of the contact of nanofibers with the solvent (phosphate buffer) on the lysis of *Micrococcus lysodeikticus*. Results are expressed in percentages by the difference in absorbances at 450 nm. (1) PVA Nanofibers; (2) PVA/CS 90:10; (3) PVA/CS 70:30; (4) Positive (Lys) and Negative Controls in the buffer. SD (Standard Deviation) was ±0.01, and each point of the graphic was carried out in triplicates.

Figure 6A shows the cumulative release profiles as a function of time. The release pattern does not seem to be influenced by the polymers used, showing similar profiles between PVA fibers, PVA/CS 90:10 and PVA/CS 70:30. On the other hand, fibers with PBA acid crosslinks show a controlled release profile over time, with a moderate release up to 30 min [about 10% (*w*/*w*)]. Beyond 30 min, a highlighted release of the enzyme was observed until 48 h or 24 h for PVA or PVA/CS nanofibers, respectively. The presence of magnetic particles does not appear to influence the Lys release profile. In contrast, nanofibers without the crosslinks release Lys slowly, not showing a controlled release profile over time.

To study the enzymatic activity during the prolonged release assay, after reading by spectrophotometry (UV 260 nm), the samples collected during the Lys release assay from PVA and PVA/CS nanofibers 90:10 and 70:30, a solution of *M. lysodeikticus* was added to all samples, for about 8 min, to estimate the enzymatic activity as a function of the microbial lysis rate (Figure 6B).

The lysis profiles of *M. lysodeikticus* in contact with the Lys released from the different nanofibers were tested over time to confirm the results observed in the release assay. Solutions in contact with fibers with PBA acid crosslinks showed microbial lysis activity from 30 min, 1 h, and 48 h for PVA/CS 70:30, PVA, and PVA/CS 90:10 nanofibers, respectively. Contrary to the non-crosslinked fibers that show Lys release and antimicrobial activity from the first minutes, with a release profile similar to the free enzyme (Figure 6), the presence of magnetic particles appears not to influence both the release and the antimicrobial enzymatic activity.

Interestingly, the fibers without Lys, namely the PVA/CS nanofibers in both proportions tested, showed some antimicrobial activity (on average 5% of the microbial lysis).

### 2.4. In Vitro Assays with Human Colon Adenocarcinoma (CaCo-2) Cell Line

The efficiency of the foreseen biosystems (PVA + LYS + PBA and PVA + LYS + PBA + ONPs) against Caco-2 cells, used as a model of colon cancer cells, was evaluated by placing them in contact for 10 days with cells. Caco-2 cell viability was evaluated using the MTT assay, and cells were visualized using SEM.

To assess the cytotoxicity of Lys and the studied boronic acids (PBA, fPBA, and aPBA), various concentrations of these components were tested in an in vitro assay with Caco-2 cells. The viability of Caco-2 cells after one week of incubation in the presence of these components and also when exposed to an aqueous mixture of Lys and boronic acids was evaluated according to the CCD matrix design.

Statistical significance compared with the control group (Caco-2 cells) was considered for * *p* < 0.05, ** *p* < 0.001 through the One ANOVA and post-Tukey tests, and the r^2^ values represented the fit of the line to different experimental data. All boronic acids are cytotoxic to Caco-2 cells, mainly at concentrations above 0.313 mg/mL (*p* < 0.001). The fPBA acid showed the lowest rate of cell viability for all concentrations tested, showing about 70 ± 8.37% cell viability for the minimum concentration tested (0.005 mg/mL; *p* < 0.005) and an IC50 of 3.22 mg/mL (*r*^2^ = 0.9644) for Caco-2 cells after 7 days of incubation. PBA acid showed a statistically significant difference in cell viability for concentrations less than 0.039 mg/mL and greater than 0.313 mg/mL, with an IC50 of 8.18 mg/mL (*r*^2^ = 0.8203). In contrast, aPBA acid presented statistically significant values for concentration values greater than 0.078 mg/mL, with an IC50 of 7.10 mg/mL (*r*^2^ = 0.915). They showed cytotoxicity values with statistical significance about the negative control for values greater than 1 mg/mL and less than 1.0 mg/mL, while for all concentrations of Lys tested, there was a reduction in cell viability with an IC_50_ of 13.27 mg/mL.

The cell viability of Caco-2 for the mixture of Lys with boronic acids at concentrations referring to the design of the RSM matrix showed similar results to those obtained with each boronic acid individually, while Lys combined with boronic acids showed a cumulative effect as a reduction in the rate of cell viability.

**Figure 7:**
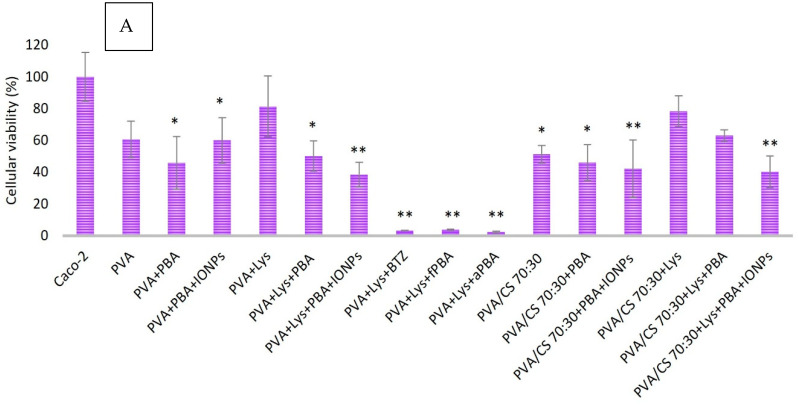

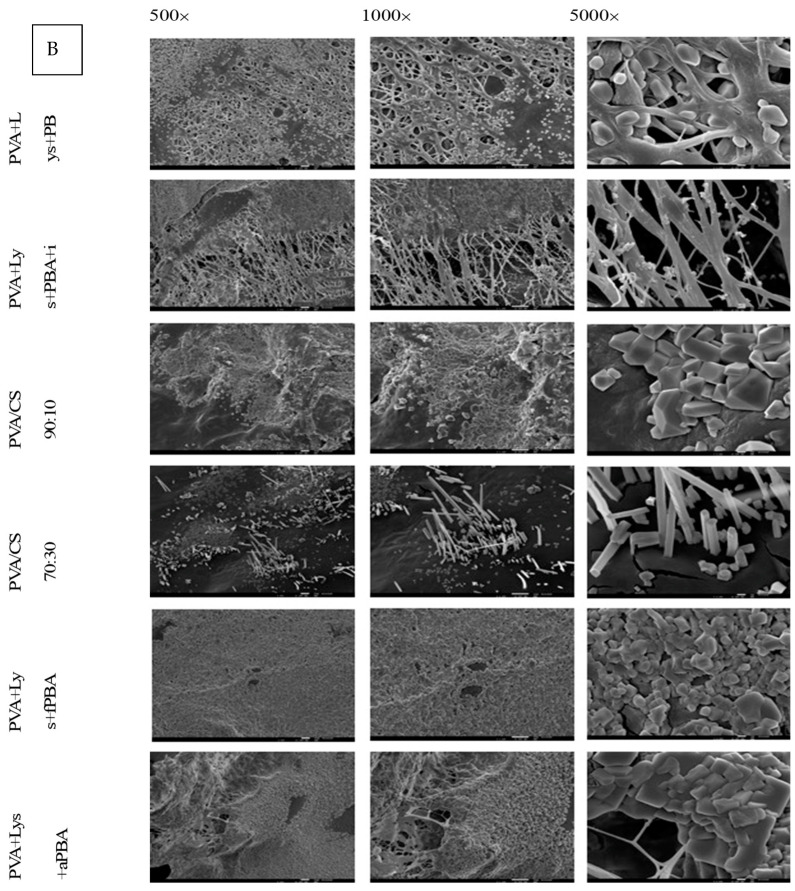
(**A**) Caco-2 cells viability after incubation with PVA and PVA/CS nanofibers by the MTT method (595 nm) at 37 °C for 7 days. Statistical significance compared with the control group (Caco-2). Statistical significance compared with the control group (Caco-2). * *p* < 0.05, ** *p* < 0.001 through One ANOVA and post Tukey tests. All data are expressed as mean ± standard deviation, *n* = 3; (**B**) Electron microscopy images of PVA + Lys + PBA nanofibers; from PVA + Lys + PBA + IONPs; PVA/CS 90:10; PVA/CS 70:30; PVA + Lys + fPBA and PVA + Lys + aPBA. After incubation with Caco-2 cells for 7 days. Magnifications of 500×, 1000× and 5000×.

This study reports various types of nanofibers, including magnetized ones, and evaluates their cytotoxic effects on colon cancer cells. The impact of nanofibers produced with lysozyme and different gel polymers was evaluated on the cell viability of Caco-2 (Figure 7). MTT results indicated a reduction in cell viability for all nanofibers tested, regardless of the treatment or polymer used. The presence of the polymers studied showed cytotoxic activity in Caco-2 cells.

Magnetized nanofibers, specifically PVA + Lys + PBA + IONPs and PVA/CS (70:30) + Lys + PBA + IONPs, exhibited high cytotoxicity compared with non-magnetized nanofibers. The cytotoxicity was observed to be significantly lower in non-magnetized nanofibers, such as PVA + Lys + PBA and PVA/CS (70:30) + Lys + PBA. Crosslinking treatment with different boronic acids resulted in a reduction in cell viability. A comparison of boronic acids (fPBA and aPBA) with positive control (crosslinking with BTZ acid) showed a similar effect on cell viability (<4%, *p* < 0.001).

Blending of polymers PVA and CS in nanofiber formation had a higher cytotoxic effect compared with PVA alone (*p* < 0.05). Figure 7B presents electron microscopy images of nanofibers used in in vitro assays with Caco-2 cells. Polyhedral structures were visible on all samples, with varying densities on different fibers. The structure of the PVA/CS 70:30 nanofibers showed “prismatic needles” that were distinct from other fibers, suggesting the possibility of Caco-2 cell adhesion. The structure of the polymeric nanofiber was noted to lose homogeneity and definition after incubation (Figure 7).

MTT results show that the nanofibers reduced the viability of Caco-2 cells. Fibers crosslinked with PBA and coated with IONPs have relatively high cytotoxicity comparable with bare PVA and PVA/CS fibers. This profile could favor the potential use of nanofibers as a co-adjuvant therapy for colon cancer.

The nanobiosystems of PVA and PVA/CS were successfully built using electrospinning. The nanofibers containing lysozyme encapsulated were produced, with increased stability by crosslinking with boronic acid derivates. Lysozyme was released gradually in different conditions of temperature and pH, namely in an acidic environment, simulated as a tumor microenvironment. The nanofibers with lysozyme encapsulated, crosslinked with fluorophenylboronic acid, and with IONPs adsorbed were able to reduce the viability of Caco-2 cells seeded on them.

Using a self-assembled nanostructured hen egg white lysozyme [35], it was able to induce 95% cell death in 24 h on MCF-7 breast cancer cells, mainly by inducing oxidative stress. The spherical nanosystem used by [35] consisted of partly folded monomeric lysozyme crosslinked with glutaraldehyde and functionalized with folic acid and was found to be stable at pH 7.4 and resistant to proteinase-K degradation. However, due to the preparation process, lysozyme was found to have lost part of its biological activity, meaning that cell death was not dependent on its enzymatic activity [35].

In vitro and in vivo studies confirmed the tumor-inhibitory activity of lysozyme. Examples of these human tumors include the uterus, colon, rectum, vulva, oral cavity, stomach, prostate, mammary carcinoma, lung carcinoma, small bowel reticulosarcoma, and multiple myeloma [36,37,38,39]. Different routes of lysozyme administration were tested, including mixing with tumor cells, peritumoral and intratumoral treatments, or indirectly through the systemic and oral routes [38,39]. The activity was dependent on the origin of the lysozyme, the type of tumor, and its degree of immunogenicity, with a potential effect on tumor lines that metastasize, suggesting that lysozyme influences the process of neoplastic dissemination [38].

One hypothesis of the antitumoral activity of lysozyme can be related to the bactericidal enzymatic action in the release of immunogenic substances, such as peptidoglycans, responsible for immunopotentiation and, consequently, antitumor activity [38,39,40].

Mahanta et al. [35] prepared, using the desolvation technique, a nanostructured self-organized lysozyme, which showed strong antiproliferative activity when tested in vitro against MCF-7 cells (breast cancer cell lines). When the antiproliferative activity of recombinant human lysozyme was tested on different stomach cancer cell lines (MGC803, MKN28, and MKN45), they showed positive results in inhibiting tumor evolution at concentrations of 100 and 1000 mg/L [39].

Wang et al. 2016 evaluated the effect of this enzyme on human lung carcinoma cells (A549 cell line) and observed that silencing lysozyme expression inhibits the invasiveness and migration of A549 lung carcinoma cells, suggesting that lysozyme is probably involved in the progression and metastasis of lung carcinoma, as a possible biomarker in the progression, prognosis and therapeutic effect of the disease.

## 3. Conclusions

Based on this study, some main conclusions can be addressed. Biodegradable nanofibers, with cytotoxic activity against Caco-2 cells, were produced adsorbed on magnetic nanoparticles, which make the system bio-responsive at higher temperatures, suggesting a potential in situ application in the treatment of tumors capable of metastasis, acting on the inhibition/reduction of the process.

## 4. Materials and Methods

### 4.1. Materials and Cells

The reagents used in this study were: poly(vinyl alcohol) (PVA, 99% degree of hydrolysis; average molecular weight 4441; Sigma Aldrich, Saint Louis, MO, USA); chitosan (CS, low molecular weight, Sigma Aldrich, Saint Louis, MO, USA); acetic acid (≥99%, VWR, Darmstadt, Germany), lysozyme obtained from hen’s egg white (Lys, ~70,000 U/mg, Sigma Aldrich, Saint Louis, MO, USA), phosphate buffer [potassium dihydrogen phosphate (KH_2_PO_4_, Merk, Darmstadt, Germany) and di-potassium hydrogen phosphate (K_2_HPO_4_, VWR, Darmstadt, Germany)], phenylboronic acid (PBA, ≥97%, Sigma Aldrich, Saint Louis, MO, USA), 2-acetyl-phenylboronic acid (aPBA, 97%, Alfa Aesar, Kandel, Gemany), 2-formyl-phenylboronic acid (fPBA) and bortezomib (BTZ); RPMI-1640 medium (Sigma Aldrich, Saint Louis, MO, USA); fetal bovine serum (PBAS, VWR, Darmstadt, Germany); 100× antibiotic/antimycotic solution (Sigma Aldrich); 10× trypsin-EDTA (Sigma Aldrich, Saint Louis, MO, USA); Hank’s Balanced Salt Solution (HBSS, Sigma Aldrich, Saint Louis, MO, USA); MTT [3-(4,5-dimethyliazol-2-yl)-2,5-diphenyltetrazolium bromide; Sigma Aldrich, Saint Louis, MO, USA) and dimethylsulfoxide (DMSO, Sigma Aldrich, Saint Louis, MO, USA).

Lyophilized *Micrococcus lysodeikticus* (*M. lysodeikticus*, ATCC No. 4698, Sigma-Aldrich, Saint Louis, MO, USA) was used.

A human colon adenocarcinoma cell line (Caco-2, ATCC No. HTB-37, Saint Louis, MO, USA) was used for cytotoxic evaluation.

All aqueous solutions were prepared with Milli-Q water.

### 4.2. Development of Electrospinning Polymer Solutions and Lys Loading

The gel polymers, PVA and CS, were used in the production of nanofibers by electrospinning.

According to the protocol described by Nunes et al. 2016 [27], aqueous solutions of PVA 10% (*m*/*v*) were prepared at about 90 °C for about 30 min under continuous magnetic stirring until complete dissolution of the polymer to a translucent gel and were subsequently stored at 4 °C.

For the CS gel solutions, a concentration of 2% (*m*/*v*) dissolved in 2% acetic acid was used, according to [28].

A solution-blending technique was employed in the production of nanofibers with both PVA-CS gel polymers. The aforementioned solutions were added in proportions 90/10 and 70/30 (*v*/*v*) with the aid of a 1 mL syringe and subsequently placed in magnetic stirring at 40–50 °C until complete dissolution of the polymers occurred.

Lysozyme immobilization was carried out at room temperature by adding the solid enzyme to the PVA or PVA-CS gel solutions at a concentration of 3% (*m*/*v*) under continuous magnetic stirring until complete dissolution (30–40 min).

### 4.3. Characterization of Gel Polymers Solutions

The electrical conductivity and surface tension of PVA and PVA-CS gel solutions (90/10 and 70/30) with and without lysozyme immobilization, used in the electrospinning assays, were evaluated.

#### 4.3.1. Conductivity

For this procedure, it was necessary to prepare 10 mL of each gel solution, with subsequent dilutions with distilled water in different proportions (%): 100, 50, 25, 12.5, 6.25, 3.125, 1.56, 0.78; 0.39; 0.20 and 0.10.

The electrical conductivity and temperature of the solutions were measured using the multiparameter conductivity meter PC5 (ref. MPMT-005-001, XS Instruments). According to the device’s instructions, the probe was introduced into the solution (10 mL), the solution was stabilized, and the conductivity value was recorded.

#### 4.3.2. Surface Tension

Surface tensions were obtained using the Wilhelmy plate technique with a Krüss K12 tensiometer. The method consisted of lifting the container containing the solution to be analyzed until the liquid surface came into contact with the plate. The force applied to the plate is measured by a microbalance. The surface tension was calculated using Equation (1):

γ = F/(ρ cosθ) (1)

where F is the force acting on the balance, ρ is the wetted length of the plate, and θ is the contact angle. The platinum plate was washed with water and acetone and flame-dried before each measurement.

### 4.4. Production of Nanofibers by Electrospinning

#### 4.4.1. PVA Nanofibers Development

After the preparation of the polymeric solutions described in 2.2, nanofibers were produced in an in-house electrospinning system consisting of a 3.4 cm diameter plastic rotating cylindrical collector with a rotation controller motor; a continuous flow pump (New Era, NE-1000, Shropshire, UK) and a potential difference generator (73030DC, Genvolt, Shropshire, UK) with up to 30 kV.

All tests were performed under the following conditions: the plastic syringe (PIC, 1 mL) was filled with the solution to be tested, and a needle (Terumo, 23 G, 0.6 × 25 mm) was connected. They were fixed to the flow pump at a distance of 8 cm from the collector and, about 1 cm from the needle tip, the positive electrode of the potential difference generator was fixed. The applied electrical potential was 17.4 kV. The nanofibers were collected in the cylindrical collector previously lined with aluminum foil, with a rotation speed of 300 rpm. The flow rate varied between 50–70 μL/min, taking into account the most suitable conditions for each solution, namely in terms of viscosity (by extrapolation). The electrospinning process took place at a room temperature of 17–19 °C.

After the formation of the nanofibers, they were carefully removed with the aluminum foil base of the collector and placed in an oven at 50 °C for 15 min. Afterward, they were stored at room temperature. The encapsulation efficiency of lysozyme in the nanofibers was 95%.

#### 4.4.2. Crosslinking and Magnetization

To improve the biomechanical properties of the fibers, they were crosslinked with boronic acids. This process includes the immersion of previously dried nanofibers in an aqueous boronic acid solution for ≈10 min. Subsequently, the nanofiber was removed and dried in an oven at 50 °C for 30–40 min. The boronic acids tested in this study are described in Supplementary Materials (Table S1).

#### 4.4.3. Iron Oxide Nanoparticles (IONPs)

The effect of magnetization of the nanofibers after crosslinking with different boronic acids was evaluated. This process consisted of immersing the nanofibers in an aqueous suspension of 2 mg/mL of iron (II, III) oxide for 1 h with stirring (40 osc/min). Then, they were removed from the aqueous suspension, dried in an oven at 50 °C for 30–40 min, and stored at room temperature.

#### 4.4.4. Characterization

The morphology of the nanofibers was evaluated using optical microscopy (OM) and electron microscopy (SEM). For SEM observation, 0.5 × 0.5 cm squares of nanofibers obtained in the different procedures were cut randomly and analyzed. The nanofibers were further characterized using a scanning electron microscopy-field emission gun (FEG-SEM) with a JEOL microscope, model JSM-7001F, operating at 5.0 kV. The surfaces were previously sputter-coated with a gold layer 20 nm thick to avoid charging effects during observation. Some fibers were cut to expose the interior.

### 4.5. Release and Enzyme Activity Assays

The Response Surface Methodology (RSM) is a statistical tool widely used in the modeling and optimization of various biotechnological processes based on factor analysis, which aims to obtain the maximum information about a process with a reduced number of trials [41]. One of the matrices used to determine quadratic models in RSM is the Central Composite Design (CCD). The design of a CCD matrix includes a 2 k factorial part, with two levels (minimum, −1 and maximum, 1) for each factor, these points being represented by (±1,…, ±1); an axial part (2 k points), at levels ± α (with α = √2) generally represented by (±α,…, 0) and (0,…, ±α); and central points, represented by (0,…, 0).

In order to study the influence of pH and temperature on the release of Lys immobilized on the nanofiber, a CCD matrix was designed with 2 factorial levels and triplicate of the central point, making up 11 tests: 22 factorial points; 3 repetitions of the central point and 4 axial points, positioned at a distance α from the central point, with α = 1.147. The pH and temperature values were defined based on previous studies. The range tested for pH was 6.15 (−α) to 7.85 (+α), and temperature of 23.5 °C (−α) and 50 °C (+α) (Supplementary Materials, Table S2). 

The release of Lys from the nanofibers was evaluated by its activity on a culture of *M. lysodeikticus*. In each well of a 24-microplate (Orange Scientific, Braine-l’Alleud, Belgium), the nanofiber of PVA + Lys + PBA + IONPs was placed and submerged in 2 mL of a 2.4 mg/mL solution of *M. lysodeikticus* in phosphate buffer at different pH values and temperatures according to the experimental design described in Supplementary Materials (Table S2), for 60 min, at 200 rpm. At 5, 10, 15, 30, and 60 min, a 100 mL sample was removed. All samples were read in the spectrophotometer at 450 nm for 7 min in order to evaluate the lysis of *M. lysodeikticus* and consequently deduce the enzymatic activity. As a positive control, a free Lys solution at a concentration of 0.0125 mg/mL was used instead of the nanofiber, and the negative control was only phosphate buffer or PVA + PBA + IOPNs (without) nanofiber in contact with the *M. lysodeikticus* solution.

In order to obtain a release profile over time (168 h) in the different types of nanofibers produced, and under physiological conditions of pH and temperature, 7.4 and 37 °C, respectively. The following test was carried out: 0.5 × 0.5 cm of the nanofiber was cut out and placed in a 1.5 mL Eppendorf with 500 mL of phosphate buffer. Subsequently, it was placed in an oven at 37 °C, and 100 mL samples were collected at times 5, 10, 20, 30, 60, 120 min and 24, 48, 72, 96, and 168 h. The volume removed from each sample was replaced with phosphate buffer at 37 °C, with the final contact volume of the fiber being constant (500 mL). Each sample was collected on a 96-well microplate on ice to inactivate enzyme activity, and spectrophotometry at wavelengths of 260 and 280 nm was analyzed to estimate the amount of Lys over time. After UV reading, the physiological function of the enzyme was evaluated, and 50 mL of *M. lysodeikticus* (7.2 mg/mL) in phosphate buffer pH 7.4 was added to each sample, followed by a kinetic study of the enzyme activity in a microplate reading at 480 nm for 75 cycles (±24 min) or 100 cycles (±32 min), every ±19 s. For each type of fiber, triplicates were performed. As a negative control, a sample of phosphate buffer pH 7.4 was used. The positive control for kinetic evaluation was a solution of free Lys at a concentration of 3 mg/mL in phosphate buffer pH 7.4.

### 4.6. In Vitro Assay with Human Colon Adenocarcinoma (CaCo-2) Cell Line

#### 4.6.1. Cell Culture

According to the protocol described by Frade et al. [29], the Caco-2 (human colorectal adenocarcinoma) cell line from ATCC (American Type Culture Collection) was cultured in RPMI-1640 medium supplemented with L-glutamine, 10% FBS (Fetal Bovine Serum) and 1% 100× antibiotic/antimycotic solution in 75 cm^2^ cell culture flasks. Cultures were maintained in a humidified incubator at 37 °C and 5% CO_2_ until they reached 80–90% confluence (8–10 days), approximately 10^6^ cells/mL.

#### 4.6.2. Influence of Lys, Boronic Acids, and IONPs on the Caco-2 Cell Viability

To evaluate the influence of Lys, the previously described boronic acids (PBA, fPBA, and aPBA) and the IONPs were tested on the cell viability of Caco-2. Two assays were performed: each one was tested individually in solutions of serial concentrations of Lys (0.00049–0.5 mg/mL), [IONPs] (0.00098–2 mg/mL), boronic acids (0.0049–10 mg/mL) (Supplementary Material, Table S2) and, a CCD array to assess the effect of both Lys and boronic acids both on the cell viability of Caco-2. Details about solutions of Lys, boronic acids, and IONPs at different concentrations, in distilled water are shown in Supplementary Materials (Table S3).

The CDD matrix was designed to study the combined effect of two variables: Lys and boronic acids (PBA, aPBA, fPBA, and BTZ), with a total of 11 trials, 22 trials for factorial points, triplicates of the central point, and 4 axial points, and α = 1.147 from the central point shown in Supplementary Materials (Table S3). The 11 trials were repeated 3 times for each combination of variables in order to assess the repeatability of results.

After reaching confluence, Caco-2 cells were trypsinized with 1% trypsin-EDTA (10 reagents in HBSS, diluted 1:5 in RPMI-1640 cell medium, previously supplemented with FBS and antibiotic/antimycotic solution, and plated 96 sterile wells microplates (Nunclon Surface, NuncTM), 100 mL per well. Cells were incubated with 100 mL of the compounds to be analyzed individually (Lys, boronic acids, and IONPs) in triplicate at the concentrations described previously. For the RSM assay, cells were incubated with 50 mL of each dependent variable (Lys and boronic acids) at the concentrations designated in the CCD matrix design (Supplementary Material, Table S4). The plates were incubated in a humid atmosphere at 37 °C and 5% CO_2_ for 5 days, and the cell culture was monitored using light microscopy. In both assays, negative controls, in triplicates, of Caco-2 cells with RPMI-1640 medium alone (control cells) were used.

Cell viability was evaluated through the MTT (3-(4,5-dimethylthiazolyl-2)-2,5-diphenyltetrazolium bromide) assay, a colorimetric test, which allowed measuring the number of viable cells after their incubation. First, the medium was carefully removed from each well, 200 mL of 1 mg/mL MTT solution in HBSS was added, and the plates were placed to incubate under the same conditions for 3–4 h. The medium was removed, and cells were washed with HBSS (100 mL per well). Finally, 100 mL of DMSO was added, which dissolved the purple formazan crystals. The plates were read on a spectrophotometer at 595 nm, and cell viability was calculated using the ratio (Equation (2)):

% cell viability = (Abs 595 nm treated cells)/(Abs 595 nm control cells) × 100 (2)

#### 4.6.3. Effect of PVA and PVA/CS Nanofibers on Caco-2 Cell Viability

In addition to the in vitro assays with Lys, boronic acids and IONPs in their free form (in solution) were also tested when immobilized on PVA or PVA/CS nanofibers. In this assay, nanofibers, previously produced and treated as described previously, were cut into approximately 0.5 × 0.5 cm squares and placed in 24-well cell culture plates (Orange Scientific). Subsequently, the fibers already on the plates were sterilized by UV for 20 min. After the confluence of the Caco-2 cell culture, they were trypsinized and diluted 1:3 in RPMI-1640 cell medium, previously supplemented with FBS and antibiotic/antimycotic solution, and transferred (500 mL) directly on top nanofibers, and the cultures were incubated in a humid atmosphere, 37 °C, and 5% CO_2_ for 7 days. During the incubation time, the culture was monitored. On day 7 of the incubation, the fibers were removed to a new plate, the culture medium was removed, and the cell viability was evaluated using the MTT assay in the plates with only cells. In this process, 500 mL of MTT and 200 mL of DMSO were used. The plates that only contained the fibers removed from the cell incubation were later observed using electron microscopy to assess whether there was adhesion and proliferation of cells in the fibers.

### 4.7. Statistical Analysis

All data referring to in vitro assays were repeated at least 3 times. The representativeness of these data was presented by the mean ± standard deviation. Statistical analyses were performed using IBM SPSS Statistics, version 24.0 55, considering a significance level, α, of 0.05.

## Figures and Tables

**Table 1:** Critical aggregation concentration (c.a.c.), specific conductivity, and surface tension at c.a.c. for each polymer solution.

Polymeric Solution	κ ^1^ (S cm^−1^)	c.a.c._κ_^2^ (%)	γ ^3^ (mN m^−1^)	c.a.c._γ_^4^ (%)
PVA 10% (*m*/*v*)	985	1.07	38.86 ± 0.40	0.68
PVA 10% (*m*/*v*) + Lys	1958	0.03	36.70 ± 0.36	2.27
CS 2% (*m*/*v*)	2324	----	----	----
PVA/CS 90:10 (*v*/*v*)	1503	2.62	37.92 ± 0.34	2.54
PVA/CS 90:10 (*v*/*v*) + Lys	1958	2.69	35.25 ± 0.30	2.48
PVA/CS 70:30 (*v*/*v*)	1466	2.49	42.35 ± 0.37	2.43
PVA/CS 70:30 (*v*/*v*) + Lys	1943	2.60	41.31 ± 0.31	2.62

^1^ Specific conductivity of the solution at the concentration that was used in electrospinning. ^2^ Critical aggregation concentration from conductivity measurements. ^3^ Surface tension of polymer solutions diluted 1:2 in distilled water. ^4^ Critical aggregation concentration from surface tension measurements.

## Data Availability

The data presented in this study are openly available in article.

## Supplementary Materials

The following supporting information can be downloaded at https://www.mdpi.com/article/10.3390/gels9120968/s1, Figure S1: Nanofibers. (A) PVA; (B) PVA/CS 70:30 + Lys; (C) PVA + Lys + PBA + IONPs; (D) PVA/CS 90:10; Figure S2: Optical microscopy (400× magnification) and electron microscopy (500× magnification) images of the nanofibers of: PVA (A); PVA + PBA (B); PVA + PBA + Lys (C); PVA + Lys (D); PVA + Lys + PBA (E); PVA + Lys + PBA + IONPs (F); PVA + Lys + aPBA (G); PVA + Lys + fPBA (H); PVA + Lys + BTZ (I); PVA/CS 90:10 (J); PVA/CS 90:10 + Lys (K); PVA/CS 90:10 + Lys + PBA (L); PVA/CS 70:30 (M); PVA/CS 70:30 + Lys (N); Table S1: Boronic acids used in the crosslinking process; Table S2: CCD matrix delineation of the effect of pH and temperature on releasing Lys from the nanofiber; Table S3: CCD matrix design of the effect of blending Lys and boronic acids (PBA, aPBA, fPBA and BTZ) on the cell viability of Caco-2; Table S4: showed the different concentrations of lysozyme (Lys), boronic acids and IONPs tested on the Caco-2 cell viability.
