# Supplementary material for: Exploring Functionalized Magnetic Hydrogel Polyvinyl Alcohol and Chitosan Electrospun Nanofibers
Source: Gels. 2023 Dec 11;9(12):968. doi: 10.3390/gels9120968 (PMC10743178; doi:10.3390/gels9120968)
Supplement: Supplementary file 1 [file gels-09-00968-s001.zip › gels-2730386-supplementary.pdf]

Supplementary Materials

Exploring functionalized magnetic hydrogel polyvinyl alcohol and chitosan electrospun nanofibers

Mónica Guerra[1], Fábio F.F. Garrudo[2], Célia Faustino[1,3,4], M. Emilia Rosa[5] and Maria H. Ribeiro[1,3,4*]

Table S1 describes the boronic acids tested in this work.

Table S1. Boronic acids used in the crosslinking process.

Nomenclature IUPAC	Abbreviation	Chemical structure	Concentration tested (% m/v)
Phenylboronic acid	PBA	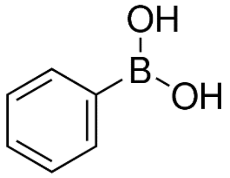	1
2-Acetyl-phenylboronic acid	aPBA	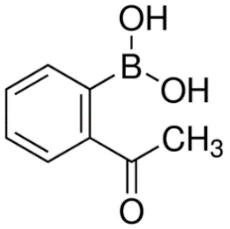	0.5
2-formyl-phenylboronic acid	fPBA	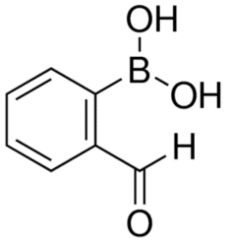	0.5
[(1R)-3-Methyl-1-[[(2S)-3-phenyl-2-(pyrazine-2-aminocarbonyl)propanoyl]amino]butyl]boronic acid	BTZ	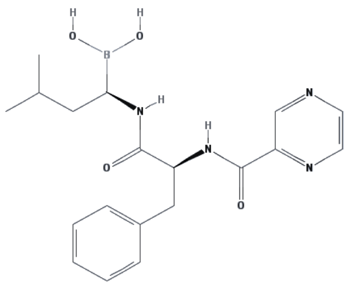	0.5

IUPAC nomenclature Abbreviation Chemical structure Tested concentration (% m/v)

Table S2. CCD matrix delineation of the effect of pH and temperature on releasing Lys from the nanofiber.

Assay	Coded values		
	pH	Temperature (ºC)	
1	7.60 (+1)	23.5 (-1)	Factorial points
2	6.40 (-1)	23.5 (-1)	
3	7.60 (+1)	42.5 (+1)	
4	6.40 (-1)	42.5 (+1)	
5	6.15 ($-\alpha$)	36.5 (0)	Axial points
6	7.85 ($+\alpha$)	36.5 (0)	
7	7.0 (0)	23.5 ($-\alpha$)	
8	7.0 (0)	50.0 ($+\alpha$)	
9	7.0 (0)	36.5 (0)	Central points
10	7.0 (0)	36.5 (0)	
11	7.0 (0)	36.5 (0)	

Table S3. CCD matrix design of the effect of blending Lys and boronic acids (PBA, aPBA, fPBA and BTZ) on the cell viability of Caco-2.

Assay	Coded values		
	Lys (mg/mL)	PBA, aPBA, fPBA (mg/mL)	BTZ (mg/mL)
1	2.00 (+1)	9.76×10^{-3} (-1)	4.88 $\times10^{-3}$ (-1)
2	9.76×10^{-4} (-1)	9.76×10^{-3} (-1)	4.88 $\times10^{-3}$ (-1)
3	2.00 (+1)	20.00 (+1)	10.00 (+1)
4	9.76×10^{-4} (-1)	20.00 (+1)	10.00 (+1)
5	9.76×10^{-4} ($-\alpha$)	0.63 (0)	0.31 (0)
6	1.44 ($+\alpha$)	0.63 (0)	0.31 (0)
7	0.03 (0)	9.76×10^{-3} ($-\alpha$)	4.88 $\times10^{-3}$ ($-\alpha$)
8	0.03 (0)	14.76 ($+\alpha$)	7.38 ($+\alpha$)
9	0.03 (0)	0.63 (0)	0.31 (0)
10	0.03 (0)	0.63 (0)	0.31 (0)
11	0.03 (0)	0.63 (0)	0.31 (0)

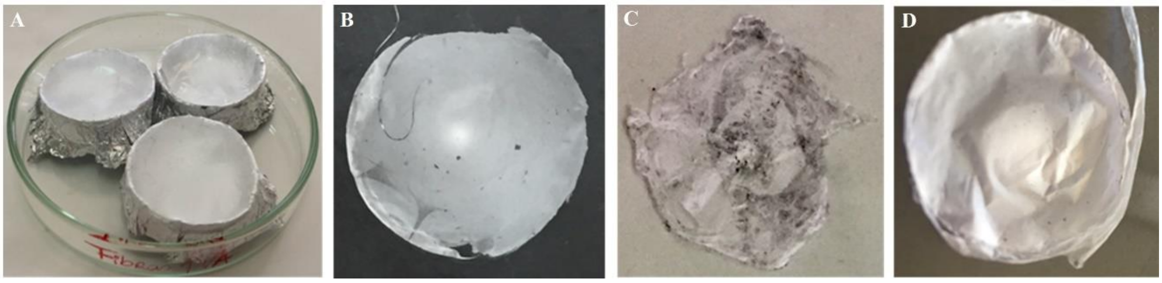

Figure S1. Nanofibers. (**A**) PVA; (**B**) PVA/CS 70:30+Lys; (**C**) PVA+Lys+PBA+IONPs; (**D**) PVA/CS 90:10.

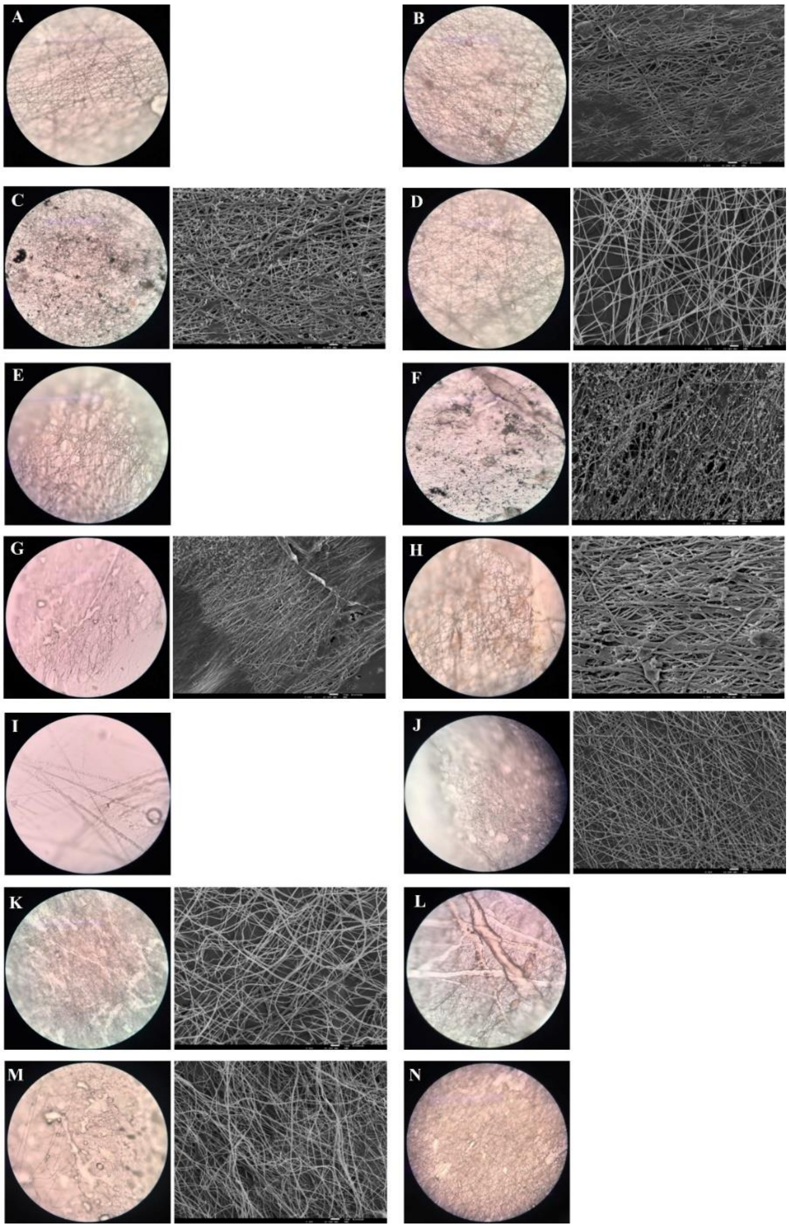

Figure S2. Optical microscopy (400x magnification) and electron microscopy (500x magnification) images of the nanofibers of: PVA (**A**); PVA+PBA (**B**); PVA+PBA+Lys (**C**); PVA+Lys (**D**); PVA+Lys+PBA (**E**); PVA+Lys+PBA+IONPs (**F**); PVA+Lys+aPBA (**G**); PVA+Lys+fPBA (**H**); PVA+Lys+BTZ (**I**); PVA/CS 90:10 (**J**); PVA/CS 90:10+Lys (**K**); PVA/CS 90:10+Lys+PBA (**L**); PVA/CS 70:30 (**M**); PVA/CS 70:30+Lys (**N**).

Table S4 showed the different concentrations of lysozyme (Lys), boronic acids and IONPs nanoparticles tested on the Caco-2 cell viability

Table S4. Initial concentrations used in the Caco-2 cell viability assay.

Dilution series	Lys (mg/mL)	[IONPs] (mg/mL)	Boronic acids (mg/mL)	
			PBA, aPBA, fPBA	BTZ
**1:1**	**1**	2	10	5
1:2	0.5	1	5	2.5
1:4	0.25	0.5	2.5	1.25
1:8	0.125	0.25	1.25	0.625
1:16	0.0625	0.125	0.625	0.313
1:32	0.0313	0.0625	0.313	0.156
1:64	0.0156	0.0313	0.156	0.0781
1:128	0.00781	0.0156	0.0781	0.0391
1:256	0.00391	0.00781	0.0391	0.0195
1:512	0.00195	0.00391	0.0195	0.00977
1:102	0.000977	0.00195	0.00977	0.00488
1:205	0.000488	0.000977	0.00488	0.00244
